# Post COVID-19 waitlist reduction in a memory disorder clinic

**DOI:** 10.3389/frhs.2025.1644087

**Published:** 2026-01-09

**Authors:** Sydney Hurt, Ian Moore, Kalpana P. Padala, Prasad R. Padala

**Affiliations:** 1 Geriatric Research Education and Clinical Center (GRECC), Central Arkansas Veterans Healthcare System (CAVHS), Little Rock, AR, United States; 2 Department of Geriatrics, University of Arkansas for Medical Sciences (UAMS), Little Rock, AR, United States; 3 Baptist Health-UAMS Graduate Medical Education, Little Rock, AR, United States; 4 Department of Psychiatry, University of Arkansas for Medical Sciences (UAMS), Little Rock, AR, United States

**Keywords:** consult management, COVID-19, dementia, geriatric, memory clinic, neuropsychology, pandemic, waitlist

## Abstract

**Introduction:**

As in-demand, specialty service providers, neuropsychologists and dementia evaluation teams in the Veterans Health Administration often face significant patient backlogs, many of which worsened during the COVID-19 pandemic. As long waitlists can result in delayed care, effective methods for reducing waitlists are essential. The purpose of this clinical quality improvement (QI) project was to increase clinical efficiency by implementing comprehensive criteria to streamline consult management in an interdisciplinary memory disorder clinic within the Central Arkansas VA healthcare system.

**Methods:**

This project used a combination of the Reach, Effectiveness, Adoption, Implementation, and Maintenance (RE-AIM) framework and the practical, robust implementation and sustainability model (PRISM) primarily for implementation purposes. Consult management criteria were developed and chart reviews utilizing these criteria were performed on all referrals to determine if patient needs could be best addressed though the memory clinic or other departments.

**Results:**

A total of 195 consults were reviewed between August 2023 and April 2024, with approximately 40% of referrals triaged to other services to appropriately address their needs. Increased administrative support and educating referring providers were also implemented. Consult tracking showed waitlist reduction from approximately 6 months to less than a month with consistent implementation and has been maintained at that level.

**Conclusions:**

Overall, implementation of our team's consult management criteria greatly improved efficiency, by reducing the clinic's wait list by prioritizing patients whose needs could be best served by our clinic while providing alternative referrals for patients whose care could be better and more expediently addressed by other services.

## Introduction

The COVID-19 pandemic had numerous adverse impacts on healthcare systems, and the Veterans Health Administration (VHA) was one among many who had to manage patient safety and quality of care during a crisis (1). Disruptions and delays in access to and utilization of different healthcare services were commonly faced concerns at both the VHA (2) and other healthcare services (3–5). Many services found themselves with an increasing backlog of patients, resulting in numerous challenges including reduced access to care and increased chance of poor patient outcomes [including elevated mortality rates (6)], as well as risk of increased financial burden due to complications related to delayed care (7). Even now, medical systems are struggling to recognize and effectively manage the backlog that accrued during that time (8–10), and the field of neuropsychology is no exception. Neuropsychology as a field took a variety of stances in response to the COVID-19 pandemic including closing clinics, reducing caseloads, or adjusting their clinical practices to include some form of teleneuropsychology (11–13).

Currently, many neuropsychologists face significant patient backlogs that only worsened during the COVID-19 pandemic, which can significantly worsen patient outcomes as delays in identification and treatment of conditions have been associated with negative consequences. Even before the pandemic, delays in diagnosis and treatment were a subject of research for the VHA to determine and address root causes for said delays (14). For neuropsychologists, timely diagnosis of neurodegenerative disorders such as dementia impacts their ability to connect affected individuals with early interventions for potentially slowing progression of the disease which has impacts on both personal and societal levels (15). Early diagnosis and intervention for neurodegenerative disorders results in improved patient adjustment, slower disease progression, economic savings, increased patient independence, and delayed need for nursing home care or hospital admission, demonstrating that timely purveyance of neuropsychological assessment services is essential for optimal patient care (16, 17).

While there has been improvement in awareness of potential risk factors as well as in detection, diagnosis, and potential prevention of dementia, it remains a growing concern as the number of individuals living with dementia is projected to increase to 152 million by 2050 (18). Notably, the prevalence of veterans with dementia is also expected to increase in the coming years (19). Furthermore, research has also highlighted differences in the incidence of dementia in older veterans depending on geographical location, with the highest incidence consistently observed in the Southeast and South, which includes Arkansas (20). Limited access to neuropsychological services, especially in rural areas (21) such as many parts of Arkansas, means that waitlists for providers and clinics can extend for several months or more.

While there was an increase in use of telehealth services for delivery of neurocognitive evaluations (11–13) which can address certain barriers to care, continued use of telehealth for neurocognitive evaluations is highly variable. Telehealth utilization is complicated by concerns related to test validity and security as well as frequent changes in coding/billing for telehealth services (21, 22). Additionally, factors such as access to and comfort with technology (23–25), level of health literacy (26), access to specialized healthcare such as neuropsychology in rural areas (21, 22) have a significant impact on service availability. The pandemic highlighted the importance of addressing barriers such as low digital literacy (27, 28) and attitudes towards use of telemedicine (29) with the hope of continued and increased utilization of these services beyond the necessity of pandemic conditions. However, use of telemedicine alone does not address the growing waitlists many providers may find themselves facing.

Prior research on reducing wait times for neurocognitive evaluations has found success with interventions such as improving scheduling practices, adjusting appointment length, and increasing inter-professional consultation (30). More generalized research on strategies for improving VHA services recommended updating policies and procedures, strengthening communication, and standardization of care to address root causes of delays in diagnosis and treatment (14). Building from that research, another potential way to reduce a clinics' backlog of referrals is through consult management processes which prioritize seeing patients most in need of neurocognitive evaluation services while simultaneously triaging patients whose care could be better addressed by other clinics/providers (31). The purpose of the present quality improvement (QI) project was to create and implement comprehensive criteria to streamline consult management in an interdisciplinary memory disorder clinic at the Central Arkansas Veterans Healthcare System.

## Methods

### Clinical setting

The interdisciplinary memory disorders clinic is a highly utilized, in-demand service that, due to available resources, is only offered one day a week in our tertiary care VA medical center. This clinic works as a one-stop assessment for cognitive impairment and associated neuropsychiatric symptoms.

### Multidisciplinary team

The clinic is staffed by neuropsychology, social work, pharmacy, geriatrics, and geriatric psychiatry. Each discipline is present at each clinic visit with patients, with full-time equivalent (FTE) allotments ranging from 0.2 for social work to 0.5 for geriatric medicine, geriatric psychiatry, and neuropsychology.

### Veterans referred

Veterans reporting cognitive difficulties are the primary referral source for this clinic. The clinic evaluates approximately 400 patients annually, although it receives substantially more referrals.

### Referral sources

Typical referral sources for this clinic include primary care providers, neurology, and general mental health providers. Consults are placed via filling out a templated referral form via the electronic health record.

### Consultation process

This consultation clinic evaluates six to eight new patients each week. Patients are evaluated by each individual provider and a consensus diagnosis and treatment plan are arrived at in an interdisciplinary manner. With the current model, patients are seen by each provider during their memory clinic appointment. Once a patient completes a portion of the evaluation with one provider, they are seen by the next provider until they have completed appointments with each discipline (social work geriatric psychiatry, neuropsychology) typically during the same day, barring extenuating circumstances. Typically the vast majority of patients are seen only once before being sent back to the referring provider (most often a primary care physician), a minority are seen for 2–3 visits based on the acuity of behavioral problem(s) associated with cognitive impairment.

### Scheduling

Once consults have been reviewed and deemed appropriate for the memory disorders clinic, they are forwarded to a Medical Support Assistant (MSA) for scheduling. Due to inconsistent funding for a dedicated MSA for the memory disorder clinic, there was inconsistent patient follow-up and delayed scheduling which greatly hampered clinic efficiency. This is particularly impactful as this is the only such clinic in the Central Arkansas Veterans Healthcare System (CAVHS) and thus in high demand. This QI project was initiated when the wait time for the clinic evaluation was over six months, changes in legislation increased the number of veterans eligible for care, and there was no comparable community care (i.e., other memory disorder clinics or similar specialized services) available.

### Intervention

**Figure 1:**
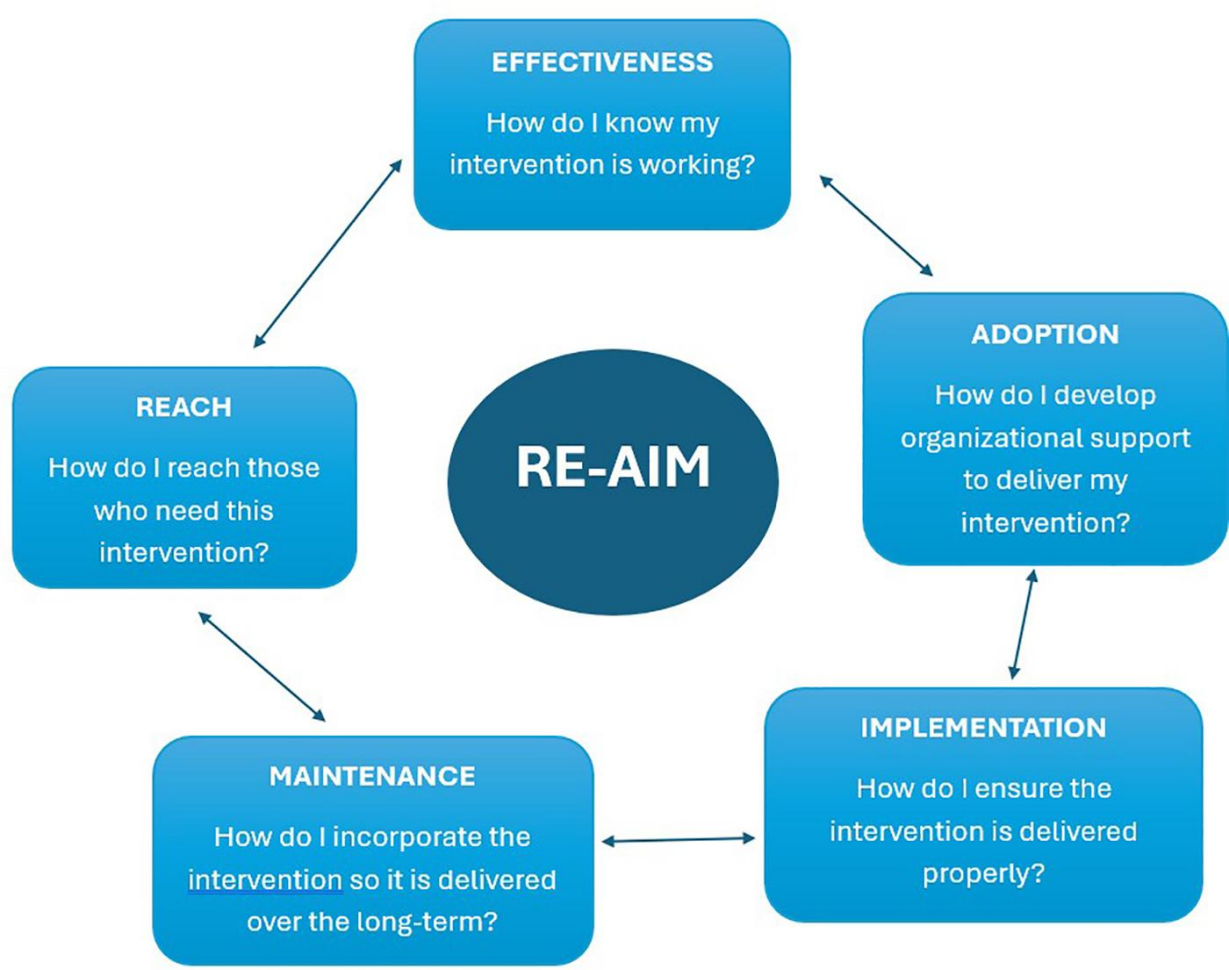
Original RE-AIM framework.

**Figure 2:**
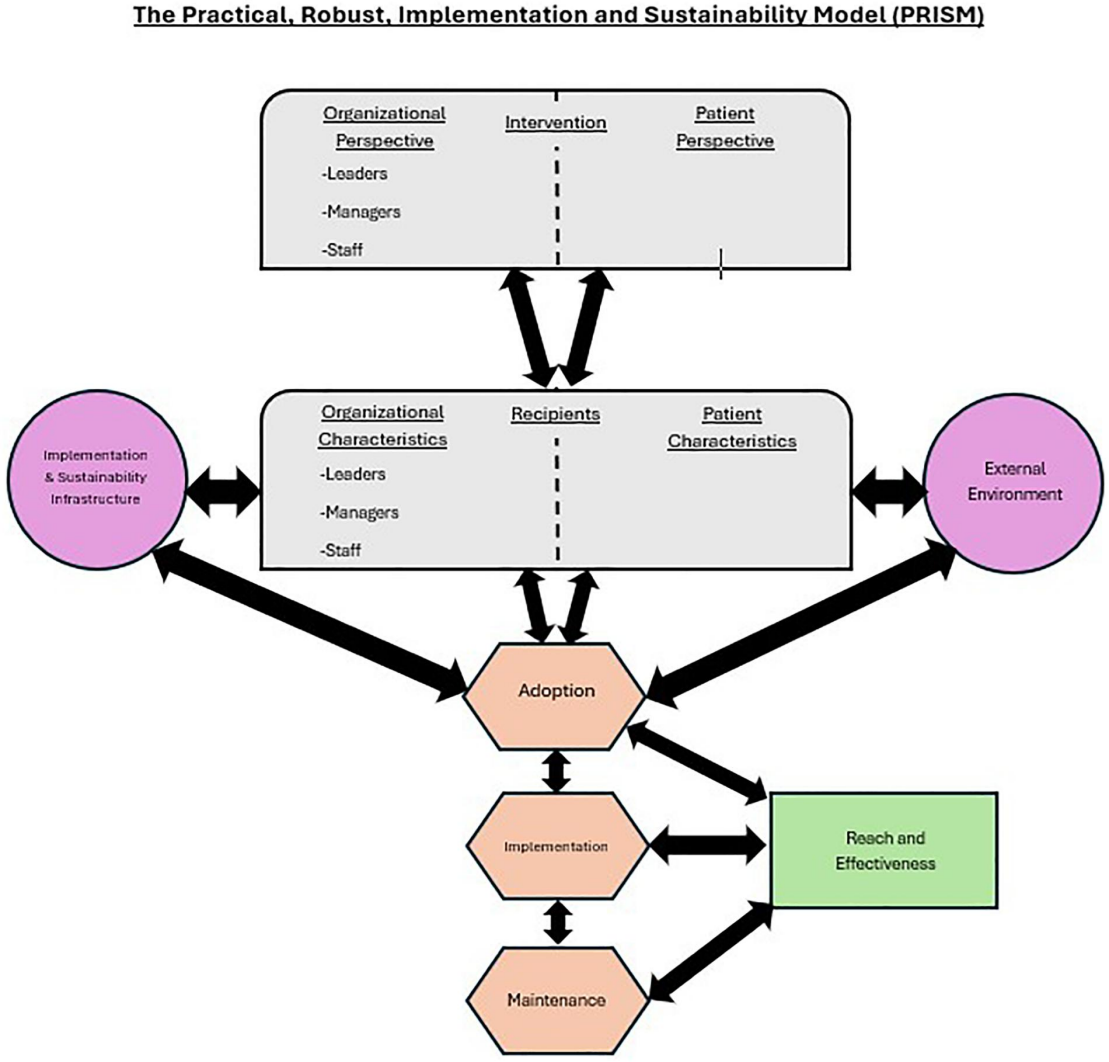
Original PRISM model.

To aid in development and implementation of a process for addressing the growing waitlist, this project utilized a combination of the Reach, Effectiveness, Adoption, Implementation, and Maintenance (RE-AIM) framework (32) which is detailed below in Figure 1 (33) and the practical, robust implementation and sustainability model [PRISM (34)]. Selected elements of the PRISM model [see Figure 2 (34)] including Intervention, Recipients, External Environment were incorporated within the RE-AIM model. Since Implementation and Sustainability Infrastructure, Adoption, Implementation, and Maintenance are shared by both the RE-AIM and PRISM models, they were not repeated in Table 1 below. This project was reviewed and approved (IRB number 699307) by the institutional review board (IRB) at the Central Arkansas VA healthcare system who determined it to be a quality improvement (QI) project.

**Table 1:** Elements of PRISM model.

Intervention	Leaders	Managers	Staff
Organizational Perspective	Assisted in development of criteria	Conducting case reviews	Need dedicated administrative assistant
Patient Perspective	Difficulty rescheduling appointments		
	Difficulty managing loved ones with agitation		
	Worsening cognitions while waiting for appointments		
External Environment	PACT & MISSION Acts caused influx of patients		
	Limited community care available		
	Suboptimal community resources		
**Recipients**	Leaders	Managers	Staff
Organizational Characteristics	Offering an alternate option of Geriatric E-consults		
Patient Characteristics	Improved access to most appropriate care		
	Right care at right time		

## Results

Prior to implementation of the QI project, almost all referrals received were accepted under the assumption that the referral met general inclusion criteria (i.e., individuals older than 65 years endorsing memory concerns, no reported active substance abuse). However, these criteria were deemed insufficient by the memory clinic team and new exclusionary criteria were agreed upon with the intention of ensuring more appropriate referrals and improving patient care and experience.

During the implementation phase of the RE-AIM framework, consult reviews were conducted between August 2023 and April 2024 for VA Memory Disorders Clinic. A total of 195 consults were reviewed by members of the memory disorders clinic, including neuropsychologists and geriatric psychiatrist, who conducted chart reviews of the electronic medical record to determine appropriateness for receiving services in the memory disorder clinic based on the new consult exclusionary criteria (see Table 2 for full criteria). Excel spreadsheets were used to record whether each consult met the exclusionary criteria and whether they were appropriate for the memory disorder clinic or should be triaged elsewhere. Approximately 40% of referrals were triaged to other services where their medical and/or mental health needs could be addressed appropriately.

**Table 2:** RE-AIM framework.

RE-AIM element	Evaluation metric	Action
Reach	# of referring providers that were provided presentations Details included in the consult referral	One-on-one meetings with medical providers making frequent referrals, presentations to shareholders on consult management criteria to improve quality of referrals
Effectiveness	Wait time from the time consult was released to first appointment (days) No-show/cancellation rates Patient satisfaction survey	Changes in overall wait-time, no-show rates, and clinic utilization rates. Feedback from patients
Adoption	# of collaborators educated about the new criteria for consults Inclusion of the new consult template in the electronic medical record	Meeting with E-Consult team, memory disorder clinic team meetings, updating current memory disorder clinic consult template to reflect new consult management exclusionary criteria (patient age <70, pre-existing dementia diagnosis, already receiving treatment, comorbid complex medical and mental health diagnoses, primarily for capacity evaluation), getting support from director of clinical care services
Implementation	# of consults that were rescheduled or resubmitted # of providers that requested additional information	Frequent communication with memory disorder clinic team members, consistent review of new and existing consults, conversations with referring providers regarding consults, review of rescheduled and resubmitted consults
Maintenance	# of monthly primary care meetings at which the new consult template was presented	Continuing education of medical providers, creation of formal standard operating procedures, orienting new team members with current consult management criteria

**Figure 3:**
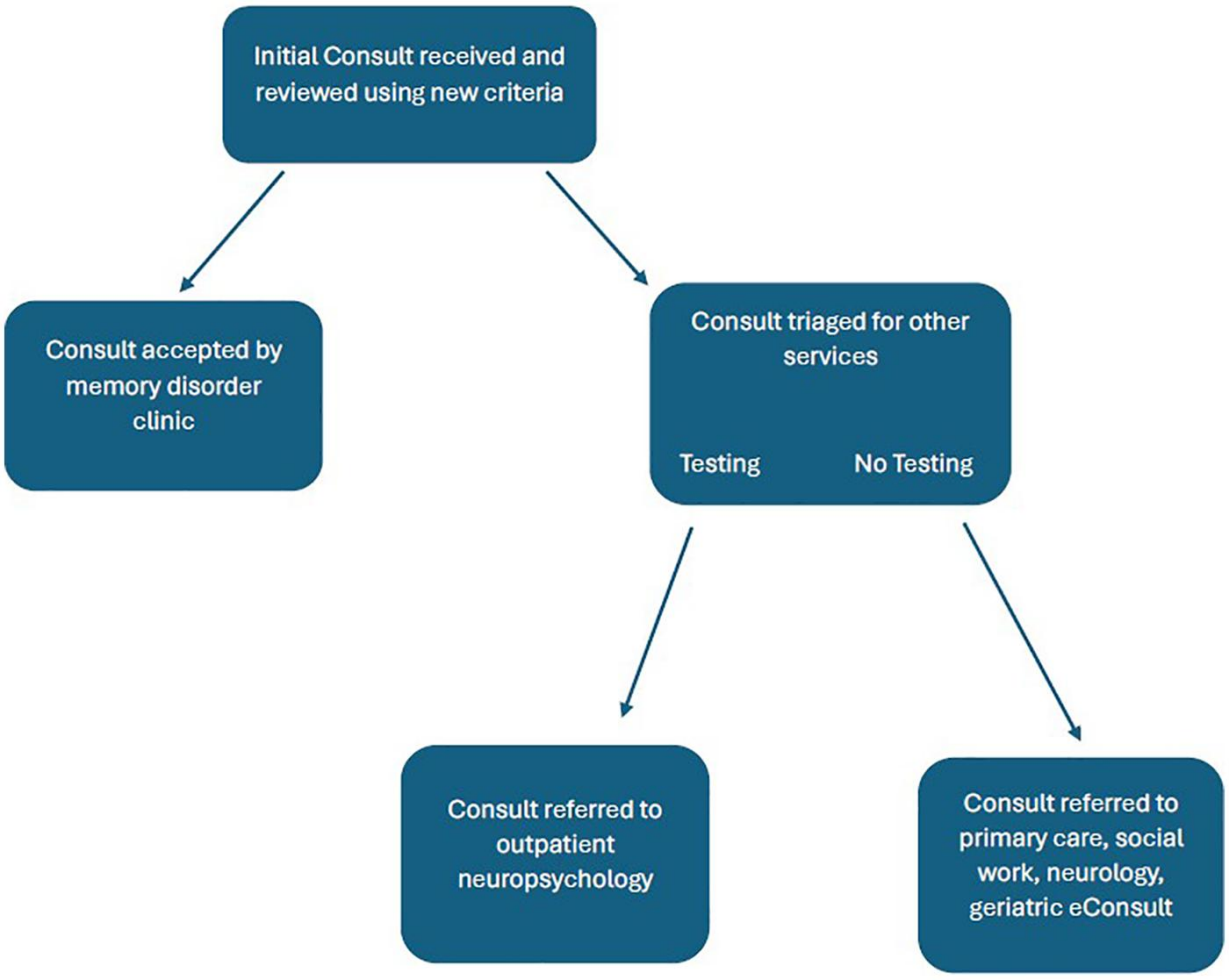
Consult flowchart.

Of referrals that were triaged, nearly 58% were deemed inappropriate for neuropsychological testing and directed to non-assessment-based care such as primary care, social work, neurology and geriatric e-consult. The remaining 42% were referred to outpatient neuropsychology as they required assessments beyond the resources of the memory disorders clinic including individuals deemed too young to be appropriately evaluated by the memory clinic and those primarily requiring a capacity evaluation. Please see flowchart (Figure 3) for additional information on referral management. Consults that contained too little information to make a determination about the best avenue of care for a veteran were addressed by requesting additional clarifying information from the referring providers about the nature of the consult request before being accepted for care by the memory disorder clinic or being triaged elsewhere as necessary.

The 58% of referrals that were triaged elsewhere for care were due to factors including: 1) referral being sent too soon following an acute brain injury/stroke to allow for adequate physical and cognitive recovery, 2) significant untreated mental health difficulties that could confound cognitive testing results, 3) profound evidence of major neurocognitive disorder diagnosis confirmed by prior evaluation already receiving medical treatment including medications, and 4) active/ongoing substance use and abuse which could also complicate patients' diagnostic pictures. At its highest caseload, the memory disorder clinic serviced up to 8 patients a day, in part in an effort to reduce the wait time patients had before accessing care. However, this approach was untenable as it drastically limited the time the team could allocate to each patient, thus potentially reducing the quality of their care. With improved consult management, the clinic has been able to reduce its caseload to 4 patients a day which has allowed for more comprehensive evaluation by neuropsychology, social work, geriatrics, and geriatric psychiatry, thus improving patient care.

Prior to this reduction in caseload, all patients in the memory disorders clinic were provided cognitive screenings by neuropsychology as well as a diagnostic interview including medication considerations by a geriatric psychiatrist or geriatrician. However, due to staffing limitations, typically only around 4 patients per day were able to be provided with social work services. The patients seen by social work were prioritized by level of need based on record review. A pharmacist was of limited on call availability due to staffing restrictions as well and was only utilized in cases where significant polypharmacy concerns were present.

By reducing the caseload via improved consult management criteria, numerous improvements in clinical care have occurred. Neuropsychology was able to provide more comprehensive testing batteries, allowing for improved diagnostic utility and more personalized treatment recommendations. Social work gained the ability to meet with and offer services to every memory disorder clinic patient due to the lower caseload. Geriatric medicine and geriatric psychiatry were able to increase the amount of time spent with patients and caregivers during their appointment, improving overall patient experience. Additionally, pharmacy could now assist with medication management for the reduced caseload rather than only in cases of polypharmacy concerns.

The memory clinic team also provided in-services to providers, ranging from sharing information in staff meetings to individual meetings with providers making frequent referrals, to educate them on the various roles and functions of the memory clinic to include the limits of care that could be provided in this setting. We utilized the GRECC “Education Bursts” model that provides brief educational material over several convenient locations and times such as in regularly scheduled staff meetings, and between patient visits for busy outpatient providers (35). Despite these educational efforts, there were a few providers who expressed additional concerns with consults being triaged and contacted the memory clinic team directly to request additional consideration of a patient for whom an evaluation was requested; infrequently patients were rebooked in the clinic after further discussions with referring providers helped elucidate the reason they were referred and how the clinic could best address their care needs.

As part of the intervention, the memory disorder clinic advocated to facility leadership for designation of a permanent MSA for the clinic from the medical center which resulted in assignment of a permanent MSA. Additionally, if needed, a memory clinic provider spoke with patients via phone call to allay anxiety or provide service recovery as needed.

Consult tracking, including compiling information about consultation receipt and completion dates, showed the clinic's waitlist was initially reduced from approximately 6 months to 3 months. With consistent implementation of new consult management criteria via chart review of each new consult, the clinic's waitlist was further reduced to less than a month from initial referral to consult completion. Owing to the increased contact with the MSA and judicious reallotment to other clinical services, longitudinal data collected via normal clinic tracking showed fewer appointment cancellations and no-shows which further increased the clinic utilization metrics. Verbal feedback received from the patients evaluated in the memory clinic was positive as well, with many patients expressing appreciation for short clinic wait times and improved communication with the team throughout the scheduling and assessment process. Discussion among memory clinic team members at monthly staff meetings indicated they generally felt that they were able to provide better care for the patients with this updated model as well due to a reduced caseload that allowed for greater time to be spent with patients.

## Discussion

Like many clinics providing a specialty service, the CAVHS memory disorder clinic developed a significant backlog due to multiple factors. Due to limited resources, the clinic is only offered one day a week. The PACT act, which expanded healthcare eligibility for veterans with presumed toxic exposures (36), led to a significant influx of patients seeking services from an already heavily utilized clinic. Additionally, the MISSION act created a veterans community care program (37) which allows them to seek care outside of the VA in an effort to increase access to timely care. However, since the memory disorders clinic is a highly specialized service, community care options were limited and those few options that were available often had similar or longer wait times. When Covid-19 guidelines suspended in-person evaluations for a significant amount of time, the waitlist grew further to approximately six months in 2023.

From a patient perspective, individuals were experiencing long wait times for care and, if rescheduling was necessary, access to care was delayed further due to the length of the waitlist. Patients who requested scheduling within a certain time frame were sometimes unable to be accommodated due to lack of clinic availability due to patient volume. Family and caregivers had to seek alternative services for dealing with loved ones experiencing worsening cognition and accompanying neuropsychiatric symptoms or attempt to manage on their own until their long-awaited appointment. To provide services to veterans and their families how and when they most needed them (i.e., “right care at the right time”), adjustments had to be made to the existing memory disorder clinic consult management process to ensure assess to timely and appropriate care.

Our QI project proved that by appropriately managing referrals and reducing the waitlist, there is an improvement in our ability to provide patients with timely, high-quality care that can most effectively address their needs based on our clinic's capabilities. A reduced waitlist has the potential to improve overall patient experience as they are getting more expedient access to care by being scheduled in the memory disorder clinic or quickly allowing their referring provider to revise their referral to more appropriately address their needs rather than having to do so after waiting potentially months for an appointment in a clinic that was incapable of doing so. By implementing this new consult management criteria into normal clinical practice via chart review of each incoming consult, this intervention has continued to be maintained as part of standard operating procedures.

The combination of RE-AIM framework and elements of PRISM provided a scaffolding for development and implementation of an intervention utilizing improved consult management criteria. Analysis of the areas of need from both an organizational and patient perspective provided multiple avenues for improvement. Implementation of consult management criteria, combined with thorough chart reviews and providing psychoeducation to referring providers, resulted in a substantial waitlist reduction, thus providing expedient access to care for patients most in need of memory clinic services while also directing patients deemed inappropriate for memory clinic care to services that could better address their needs. Giving education to providers regarding the capabilities of the memory disorder clinic and providing consistent feedback regarding the rationale for declining consults and linking these decisions to specific inclusion criteria aided in the adoption and maintenance of the new consult management criteria. Providing information on what patients the memory disorder clinic could best serve (as well as those who might benefit most from other services) empowered referring providers by giving them the opportunity to pre-emptively decide whether a consult was more appropriate for another service prior to initial review by the memory disorder clinic team, thus preventing service over-utilization.

### Limitations and future directions

#### Intervention limitations

The reviews for suitability of the referral were limited to the information readily available in the patient's medical record. Though the VA medical records often contain a great deal of information, this is not always the case. Additionally, non-VA providers do not always have access to the same level of data this study was able to use when conducting chart reviews. A potential method to address this limitation in the future could be use of a pre-intake screening designed to solicit information relevant to consult management criteria.

#### Study limitations

Results of this study are limited by absence of key data to further contextualize the impact and effectiveness of the clinic management undertaken in this project as well as more fully appreciate its impact on patient experience. In specific, future studies would benefit from (1) quantifying time spent implementing and educating referring providers on memory clinic referral criteria, (2) evaluating patient and caregiver experience before and after implementation of the clinic management criteria to gain insight into possible improvements or drawbacks in their clinic experience, (3) quantitatively assess provider experiences before and after clinic changes are made to highlight impacts on clinical care provided, (4) tracking the number of referrals received and accepted annually as well as those which provide incomplete or inadequate referral information to help appreciate the scope and impact of clinic management criteria on these areas. Addressing these limitations in future studies would greatly benefit research on such future studies on clinic management.

At this time, this QI project has also only been implemented in one clinic. Future studies could examine whether utilizing similar consult management criteria or adoption of the RE-AIM and PRISM frameworks into different clinics might yield similar results to this project. Additionally, no information was collected about the downstream effects that increased triaging to other clinics had on wait times for those services, and future studies may consider gathering this additional data. Future studies might also include information on the cost effectiveness of the intervention, as well as tracking consults from referral to completion of the appointment and documentation, as this study stopped tracking consults after they were scheduled in the clinic or triaged elsewhere.

## Conclusions

Establishing comprehensive criteria allows neuropsychologists to manage consults in a way that prioritizes patients most in need of their services while also triaging patients more appropriate for other services. Overall, this QI project demonstrated how implementation of consult management criteria in a memory disorders clinic greatly reduced the waitlist, thus providing expedient access to care for Veterans with cognitive difficulties and their families/caregivers.

## Data Availability

The data supporting the findings are available on request from the corresponding author. The data are not publicly available due to privacy or ethical restrictions.

## References

[1] Charles MA Yackel EE Mills PD Welsh DE. Veterans health administration response to the COVID-19 crisis: surveillance to action. J Patient Saf. (2022) 18(7):686–91. 10.1097/PTS.0000000000000995 35152235 PMC9524529

[2] Mills P Louis RP Yackel E. Delays in care during the COVID-19 pandemic in the veterans health administration. J Healthc Qual. (2023) 45(4):242–53. 10.1097/JHQ.0000000000000383 37039808 PMC10313724

[3] González-Touya M Stoyanova A Urbanos-Garrido RM. COVID-19 and unmet healthcare needs of older people: did inequity arise in Europe? Int J Environ Res Public Health. (2021) 18(17):9177. 10.3390/ijerph18179177 34501767 PMC8431067

[4] Lockett M Tamayo GC Schalet BD Reise SP Kimerling R. Changes in healthcare engagement during the COVID-19 pandemic. J Patient Rep Outcomes. (2025) 9(1):21. 10.1186/s41687-025-00850-z 39976772 PMC11842638

[5] Okoro O Vosen EC Allen K Kennedy J Roberts R Aremu T. COVID-19 impact on mental health, healthcare access and social wellbeing—a black community needs assessment. Int J Equity Health. (2022) 21(1):137. 10.1186/s12939-022-01743-z 36138403 PMC9493150

[6] Miller J Wey A Musgrove D Son Ahn Y Hart A Kasiske BL Mortality among solid organ waitlist candidates during COVID-19 in the United States. Am J Transplant. (2021) 21(6):2262–8. 10.1111/ajt.16550 33621421 PMC8014331

[7] Mogharab V Ostovar M Ruszkowski J Hussain SZM Shrestha R Yaqoob U Global burden of the COVID-19 associated patient-related delay in emergency healthcare: a panel of systematic review and meta-analyses. Global Health. (2022) 18(1):58. 10.1186/s12992-022-00836-2 35676714 PMC9175527

[8] Wilkinson E. UK Government urged to recognise post-COVID-19 cancer backlog. Lancet Oncol. (2021) 22(7):910. 10.1016/S1470-2045(21)00330-2 34090554 PMC8584714

[9] Aitken RJ Watters DA. Clearing elective surgery waiting lists after the COVID-19 pandemic cannot be allowed to compromise emergency surgery care. Med J Aust. (2022) 217(5):237–8. 10.5694/mja2.51672 35918077 PMC9538332

[10] Carneiro VLA Andrade H Matias L de Sousa RARC. Post-COVID-19 and the Portuguese national eye care system challenge. J Optom. (2020) 13(4):257–61. 10.1016/j.optom.2020.05.001 32711965 PMC7211682

[11] Hewitt KC Loring DW. Emory university telehealth neuropsychology development and implementation in response to the COVID-19 pandemic. Clin Neuropsychol. (2020) 34(7–8):1352–66. 10.1080/13854046.2020.1791960 32660335

[12] Zane KL Thaler NS Reilly SE Mahoney JJ 3rd Scarisbrick DM. Neuropsychologists’ practice adjustments: the impact of COVID-19. Clin Neuropsychol. (2021) 35(3):490–517. 10.1080/13854046.2020.1863473 33371799 PMC8381282

[13] Loman M Vogt E Miller L Landsman R Duong P Kasten J How to” operate a pediatric neuropsychology practice during the COVID-19 pandemic: real tips from one practice’s experience. Child Neuropsychol. (2020) 27(2):251–79. 10.1080/09297049.2020.1830962 33059534

[14] Politi RE Mills PD Zubkoff L Neily J. Delays in diagnosis, treatment, and surgery: root causes, actions taken, and recommendations for healthcare improvement. J Patient Saf. (2022) 18(7):e1061–6. 10.1097/PTS.0000000000001016 35532991

[15] Chandler JM Ye W Mi X Doty EG Johnston JA. Potential impact of slowing disease progression in early symptomatic Alzheimer’s disease on patient quality of life, caregiver time, and total societal costs: estimates based on findings from GERAS-US study. J Alzheimers Dis. (2024) 100(2):563–78. 10.3233/JAD-231166 38875031 PMC11307086

[16] Guss R. Clinical Psychology in the Early Stage Dementia Care Pathway. Leicester: St Andrews House (2013).

[17] Livingston G Huntley J Sommerlad A Ames D Ballard C Banerjee S Dementia prevention, intervention, and care: 2020 report of the lancet commission. Lancet. (2020) 396(10248):413–46. 10.1016/S0140-6736(20)30367-6 32738937 PMC7392084

[18] Alzheimer’s Association. 2024 Alzheimer’s disease facts and figures. Alzheimers Dement. (2024) 20(5):3708–821. 10.1002/alz.13809 38689398 PMC11095490

[19] U.S. Department of Veterans Affairs. Projections of the prevalence and Incidence of dementias Including Alzheimer's disease for the total veteran, enrolled, and patient populations age 65 and older (2013). Available online at: https://www.va.gov/GERIATRICS/docs/Methodology_Paper_Projections_of_the_Prevalence_and_Incidence_of_Dementias_v5_FINAL.pdf

[20] Dintica CS Bahorik AL Xia F Boscardin J Yaffe K. Regional differences in dementia incidence among US veterans. JAMA Neurol. (2025) 82(8):817–24. 10.1001/jamaneurol.2025.1536 40489088 PMC12150226

[21] McMillen TN Woolner K. The underrepresentation of board-certified neuropsychologists practicing in rural areas: a diversity issue and call for action. Arch Clin Neuropsychol. (2024) 39(7):1006. 10.1093/arclin/acae067.082

[22] Barton C Morris R Rothlind J Yaffe K. Video-telemedicine in a memory disorders clinic: evaluation and management of rural elders with cognitive impairment. Telemed J E Health. (2011) 17(10):789–93. 10.1089/tmj.2011.0083 22023458

[23] Sperling SA Acheson SK Fox-Fuller J Colvin MK Harder L Cullum CM Tele-Neuropsychology: from science to policy to practice. Arch Clin Neuropsychol. (2024) 39(2):227–48. 10.1093/arclin/acad066 37715508

[24] Ancker JS Barrón Y Rockoff ML Hauser D Pichardo M Szerencsy A Use of an electronic patient portal among disadvantaged populations. J Gen Intern Med. (2011) 26(10):1117–23. 10.1007/s11606-011-1749-y 21647748 PMC3181304

[25] Goel MS Brown TL Williams A Hasnain-Wynia R Thompson JA Baker DW. Disparities in enrollment and use of an electronic patient portal. J Gen Intern Med. (2011) 26(10):1112–6. 10.1007/s11606-011-1728-3 21538166 PMC3181306

[26] Bailey SC O'Conor R Bojarski EA Mullen R Patzer RE Vicencio D Literacy disparities in patient access and health-related use of internet and mobile technologies. Health Expect. (2015) 18(6):3079–87. 10.1111/hex.12294 25363660 PMC4417455

[27] Finkelstein R Wu Y Brennan-Ing M. Older adults’ experiences with using information and communication technology and tech support services in New York city: findings and recommendations for post-pandemic digital pedagogy for older adults. Front Psychol. (2023) 14:1129512. 10.3389/fpsyg.2023.1129512 37138998 PMC10150999

[28] Shapira S Yeshua-Katz D Goren G Aharonson-Daniel L Clarfield AM Sarid O. Evaluation of a short-term digital group intervention to relieve mental distress and promote well-being among community-dwelling older individuals during the COVID-19 outbreak: a study protocol. Front Public Health. (2021) 9:577079. 10.3389/fpubh.2021.577079 33898369 PMC8062707

[29] Aldekhyyel RN Alshuaibi F Alsaaid O Bin Moammar F Alanazy T Namshah A Exploring behavioral intention to use telemedicine services post COVID-19: a cross sectional study in Saudi Arabia. Front Public Health. (2024) 12:1385713. 10.3389/fpubh.2024.1385713 38689764 PMC11058790

[30] Cass J Fristad M Valleru J Gallup J Butter E. Improving wait times for pediatric neuropsychology services. Evid Based Pract Child Adolesc Ment Health. (2020) 5(2):128–38. 10.1080/23794925.2020.1784057

[31] Mietchen JJ Cieminski TM Kessler-Jones AM. Your clinical interview *is* data: the benefit of telehealth appointments to triage referrals made to pediatric neuropsychology. Clin Neuropsychol. (2025) 39(5):1266–85. 10.1080/13854046.2025.2456160 39851249

[32] Glasgow RE Vogt TM Boles SM. Evaluating the public health impact of health promotion interventions: the RE-AIM framework. Am J Public Health. (1999) 89(9):1322–7. 10.2105/AJPH.89.9.1322 10474547 PMC1508772

[33] Ory MG Altpeter M Belza B Helduser J Zhang C Smith ML. Perceived utility of the RE-AIM framework for health promotion/disease prevention initiatives for older adults: a case study from the U.S. Evidence-based disease prevention initiative. Front Public Health. (2015) 2:143. 10.3389/fpubh.2014.00143 25964897 PMC4410418

[34] Feldstein AC Glasgow RE. A practical, robust implementation and sustainability model (PRISM) for integrating research findings into practice. Jt Comm J Qual Patient Saf. (2008) 34(4):228–43. 10.1016/S1553-7250(08)34030-6 18468362

[35] Sullivan SC Garner KK Padala PR Padala K Taylor T Nabholz L Bursting” out of the box: process improvement for in-service education. Medsurg Nurs. (2018) 27(5):297–99.

[36] H.R. 3967—117 Congress (2021–2022) Honoring our PACT Act of 2022, H.R. 3967, 117 Cong (2022). Available online at: https://www.congress.gov/bill/117th-congress/house-bill/3967/text (Accessed August 22, 2025).

[37] McCain JS, Akaka DK, Johnson SR, S.2372—115 Congress (2017–2018) VA MISSION Act of 2018, S.2372, 115 Cong (2018). Available online at: https://www.congress.gov/bill/115th-congress/senate-bill/2372 (Accessed August 22, 2025).

